# A community health worker delivered intervention to address hypertension among adults engaged in HIV care in northern Tanzania: Outcomes from a pilot feasibility study

**DOI:** 10.1111/jch.14518

**Published:** 2022-07-27

**Authors:** Preeti Manavalan, Deng B. Madut, Lisa Wanda, Ally Msasu, Blandina T. Mmbaga, Nathan M. Thielman, Melissa H. Watt

**Affiliations:** ^1^ Division of Infectious Diseases and Global Medicine Department of Medicine University of Florida Gainesville Florida USA; ^2^ Division of Infectious Diseases Department of Medicine Duke University Medical Center Durham North Carolina USA; ^3^ Duke Global Health Institute Durham North Carolina USA; ^4^ Kilimanjaro Clinical Research Institute Moshi Tanzania; ^5^ Kilimanjaro Christian Medical University College Moshi Tanzania; ^6^ Department of Population Health Sciences University of Utah Salt Lake City Utah USA

**Keywords:** community health worker, HIV, hypertension, sub‐Saharan Africa, Tanzania

## Abstract

Current care models are inadequate to address the dual epidemic of hypertension and HIV in sub‐Saharan Africa. We developed a community health worker (CHW)‐delivered educational intervention, integrated into existing HIV care to address hypertension in persons living with HIV. A detailed educational curriculum was created with five sessions: three in‐person clinic sessions and two telephone sessions. The intervention was piloted among hypertensive adults at one HIV clinic in northern Tanzania over a 4‐week period. Primary outcomes were feasibility, fidelity, and acceptability of the intervention. Secondary outcomes included hypertension care engagement and systolic and diastolic blood pressure (SBP and DBP). Among 16 eligible participants, 14 (64% women, median age of 54.5 years) were recruited into the study, and 13 (92.9%) completed all five intervention sessions. The intervention was delivered with 98.8% fidelity to the curriculum content. Hypertension care engagement improved following the intervention. At baseline, two (15.4%) participants had seen a doctor previously for hypertension, compared to 11 (84.6%) participants post‐intervention (*P* = .0027). No participant was using antihypertensives at baseline, compared to 10 (76.9%) post‐intervention (*P* = .0016). Pre‐intervention median SBP was 164 (IQR 152–170) mmHg, compared to post‐intervention SBP of 146 (IQR 134–154) mmHg (*P* = .0029). Pre‐intervention median DBP was 102 (IQR 86–109) mmHg, compared to post‐intervention DBP of 89 (IQR 86–98) mmHg (*P* = .0023). A CHW‐delivered educational intervention, integrated into existing HIV care, is feasible and holds promise in improving hypertension care engagement and reducing blood pressure. Further research is needed to evaluate the efficacy and scale‐up of our intervention.

## 1 INTRODUCTION

Hypertension is a leading risk for cardiovascular morbidity and mortality in sub‐Saharan Africa (SSA).^1^, ^2^ Approximately one in three persons with treated HIV in SSA is also hypertensive;^3^ however, very few are aware of their diagnosis or are on antihypertensive treatment.^4^, ^5^, ^6^, ^7^ Among a cohort of hypertensive patients enrolled in HIV care in northern Tanzania, approximately one‐third had never had their blood pressure measured, 90% were not on antihypertensive treatment, and none had achieved blood pressure control.^7^ This suggests that current care models are inadequate to address the growing burden of hypertension in persons living with HIV (PLWH).

Major barriers to hypertension care among PLWH in Tanzania and similar settings include gaps in patient education and clinical training, staff shortages, high patient volumes, and clinician time constraints due to limitations in human resources.^8^, ^9^, ^10^, ^11^ In contrast, investments in HIV care in Tanzania have created a strong infrastructure that support care engagement and lead to virologic control.^8^, ^12^, ^13^ This suggests that the HIV clinic represents a platform that could be leveraged for the delivery of non‐communicable disease (NCD) services including hypertension in PLWH. However, implementing NCD care in the HIV clinical setting may be constrained by low clinician to patient ratios and siloed models of care and funding streams.

Task‐sharing is a potential strategy that addresses challenges in human resources and describes a situation where a task normally performed by a physician is transferred to a health professional with a different or lower level of training. This is an increasingly attractive approach to care in resource‐limited settings due to physician shortages and challenges with access to healthcare.^14^, ^15^ Task‐sharing has shown to improve both NCD and HIV clinical outcomes in resource‐limited settings;^16^, ^17^, ^18^, ^19^ therefore, a task‐sharing intervention aimed at hypertensive PLWH and integrated into existing HIV care holds potential to improve hypertension care. To this end, we developed an educational intervention delivered by a community health worker (CHW) and integrated into existing HIV care. The intervention aimed to address hypertension in order to: 1) improve hypertension care engagement outcomes among PLWH, 2) reduce systolic blood pressure (SBP) and diastolic blood pressure (DBP) in hypertensive PLWH, and 3) improve hypertension knowledge among hypertensive PLWH. In this study, we examined the feasibility, fidelity and acceptability of the intervention in an HIV clinic setting in northern Tanzania, and the potential impacts of the intervention in achieving the identified objectives.

## 2 METHODS

We conducted a pilot feasibility study aimed at determining the feasibility, fidelity, acceptability, and potential efficacy of an educational intervention delivered by a CHW for hypertensive adults enrolled in HIV care in northern Tanzania.

### 2.1 Setting

The study was conducted from April 1, 2019, through July 31, 2019, in a single HIV clinic (HIV Care and Treatment Center) located in a government health facility in the Moshi urban district of Tanzania. The clinic serves approximately 1200 adults (900 women and 300 men) with HIV per year. We previously found that approximately 20% of all adults enrolled in HIV care at this HIV Care and Treatment Center are hypertensive.^7^

### 2.2 Participants

An observational study was first conducted to determine the prevalence of hypertension in the HIV clinic; detailed sampling methods for this study have been previously reported.^7^ Briefly, all adults attending the HIV clinic over a 2‐month period were invited to have their blood pressure measured. Blood pressure was measured twice in the right arm with at least a 5‐minute interval between measures using an FDA‐approved automatic blood pressure monitor (Omron Healthcare, Bannockburn, Illinois, USA). If at least one of the two measurements was ≥ 140 mmHg systolic or ≥ 90 mmHg diastolic, then the participant was invited to return to the clinic within 1‐2 weeks for repeat blood pressure measurements. The study definition for hypertension included meeting any of the following criteria: 1) a self‐reported diagnosis of hypertension, 2) a single blood pressure measurement ≥ 160 mmHg systolic or ≥ 100 mmHg diastolic, or 3) two measurements at separate visits obtained at least 1 week apart ≥ 140 mmHg systolic or ≥ 90 mmHg diastolic. Contact information was collected with participant consent for those individuals who met study criteria for hypertension and who also expressed interest in participating in the pilot study. A subset of participants enrolled in the observational study were contacted for recruitment for the pilot feasibility study if they met the following eligibility criteria: 1) currently enrolled in HIV care at the study site, 2) on antiretroviral therapy (ART) for at least the past 12 months, 3) a documented suppressed HIV viral load < 1000 copies/mL, and 4) at least three of four SBP measurements obtained from the observational study that were between 150 and 180 mmHg and/or at least three of four DBP measurements that were between 100 and 110 mmHg. These specific blood pressure values were selected as eligibility criteria in order to select for individuals who might benefit from the intervention but who did not require emergent hypertension care. Participants in the observational study with an elevated blood pressure were instructed to follow up with an outpatient provider during the same day for further evaluation and management. Participants with a severely elevated blood pressure of ≥ 180 mmHg systolic or ≥ 110 mmHg diastolic were brought to the urgent care clinic by a member of the study team for urgent evaluation and management of possible hypertensive urgency/emergency.

A total of 555 adults were screened for the observational study of whom 105 met study criteria for hypertension. Based on the above eligibility criteria, 16 adults (11 women and 5 men) were eligible to participate in the pilot study. A research assistant called all eligible participants and provided a brief description of the research activities. Individuals who expressed interest met with the research assistant in a private research office at the study site to learn more about the study. The research staff read the informed consent aloud in Swahili, provided a paper copy in Swahili, and answered any questions before written informed consent was obtained.

### 2.3 Procedures

After providing consent, participants completed a baseline assessment that included a hypertension knowledge, attitudes, and practices (KAP) survey, and had their blood pressure measured. The first intervention session was delivered immediately following the baseline assessment. Contact information and preferred methods of contact were collected to schedule subsequent telephone and in‐person intervention sessions. A post‐intervention assessment was conducted 2 weeks following the last in‐person intervention session.

### 2.4 Intervention

**FIGURE 1:**
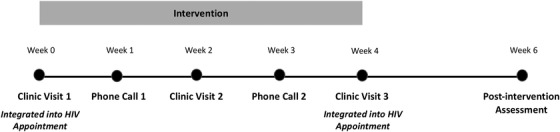
Overview of community health worker‐delivered educational intervention integrated into an HIV care and treatment center in northern Tanzania, 2019

Utilizing the Health Belief Model,^20^, ^21^ we developed an educational intervention delivered by a CHW and integrated into existing HIV clinic appointments. The Health Belief Model is a psychosocial construct that is used to explain and predict health behaviors and emphasizes certain perceptions such as perceived susceptibility of an illness, perceived severity of the illness, perceived benefits of treatment, perceived barriers to treatment, and cues to action including awareness and knowledge of the disease will lead to self‐efficacy and likelihood of adopting a behavioral change. The intervention included three in‐person sessions and two telephone sessions. A detailed educational curriculum was created for each session, based on the constructs of the Health Belief Model. Curriculum content was informed by qualitative and quantitative data collected during the observational study^7^, ^8^ and content was confirmed during the intervention pilot. The intervention was delivered to participants over a 4‐week period (see Figure 1).

Intervention session 1 focused on hypertension education, including providing an overview about the risk factors, diagnosis, symptoms, course, complications, and treatment of hypertension. The goal of this session was to create a sense of urgency to motivate the patient into action (Table 1).

**TABLE 1:** Overview of goals and content of community health worker delivered educational intervention sessions integrated into an HIV care and treatment center in northern Tanzania, 2019

**Type of intervention session**	**Content of session**	**Goal of session**	**Week of session**
Clinic session 1	Hypertension education counseling	Create a sense of urgency to motivate patient into action	0
Phone session 1	Check in and appointment reminder call	Build therapeutic relationship between patient and CHW	1
Clinic session 2	Referral to prescribing provider if indicated and counseling about antihypertensive medication	Provide patient with tools necessary to motivate behavioral change	2
Phone session 2	Check in and appointment reminder call	Identify barriers to treatment and explore ways to address barriers	3
Clinic session 3	Lifestyle modification counseling	Create long‐term commitment to behavioral change and medication use	4

Abbreviation: CHW, community health worker.

Telephone session 1 was delivered 1 week after the first in‐person session, and was a check in call to remind the patient of their next in‐person appointment and to help build a therapeutic relationship between the participant and CHW.

The second in‐person session was delivered 2 weeks after the first in‐person session and 1 week after the first telephone session. The goal of this session was to discuss use of antihypertensive medication, refer participants to a prescribing provider if their blood pressure remained ≥ 140 mmHg systolic or ≥ 90 mmHg diastolic, and to provide participants with the tools necessary to motivate behavioral change.

The second telephone session was delivered 1 week after the second in‐person session and was another check in call, with the goal of identifying initial barriers to antihypertensive treatment and to begin exploring ways to address these barriers.

The third in‐person intervention session was delivered 2 weeks after the second in‐person session and 1 week after the second telephone session. The goal of this session was to focus on lifestyle modification counseling and to foster long‐term commitment to behavioral change and antihypertensive medication use. At the end of this session, the CHW and participant consolidated the components of the intervention sessions into an individualized action plan for maintenance of hypertension care.

The CHW, a Tanzanian with secondary level education who volunteered with the local HIV advisory board, and had no prior medical training or background, was hired and trained by a physician researcher over a 2‐week period. Training sessions included instruction regarding proper blood pressure measurement techniques, general knowledge about the risk factors, causes, symptoms, diagnosis, treatment and prevention of hypertension, in‐depth review of the intervention curriculum, and role‐playing with mock intervention sessions.

At the time of this study, participants enrolled in HIV care had monthly appointments with their HIV provider in order to obtain their ART. Therefore, we attempted to schedule the first and third in‐person intervention sessions during days that participants had existing HIV appointments in order to provide an integrated, efficient and convenient model of care. All sessions were conducted in a private research room located in a building next to the study clinic. Participants who had an existing appointment with their HIV provider the same day as an intervention session had the choice of either completing the intervention session before or after their HIV appointment. In‐person intervention sessions were approximately 60 minutes in length and telephone intervention sessions were approximately 10 minutes in length.

### 2.5 Primary outcomes

Primary outcomes were feasibility, fidelity, and acceptability of the intervention.

### 2.6 Feasibility

Feasibility of the intervention was measured by the ability to recruit eligible participants into the study and to retain participants throughout all intervention sessions.

### 2.7 Fidelity

All in‐person and telephone intervention sessions were assessed for fidelity. Detailed checklists were created for each in‐person and telephone intervention session, and the checklists were used as a guide for the CHW and as a tool for the research team to assess fidelity to the curriculum content. All intervention sessions were recorded with participant consent and each session was transcribed in English. Each transcript was reviewed by two members from the research team, a Tanzanian research assistant with a bachelor's degree in public health and a US physician researcher, and using the structured checklists the recorded sessions were assessed for fidelity to the intervention. Debriefing meetings were conducted following every in‐person intervention session and also on a weekly basis. During the debriefing meetings, the team provided feedback to the CHW, discussed successes and challenges of the session, and refined the curriculum content accordingly.

### 2.8 Acceptability

At the post‐intervention assessment, all participants were asked to complete a patient satisfaction survey to provide feedback on specific aspects of the intervention, including time spent in the intervention, and perceived helpfulness of various aspects of the intervention. Participants were asked to state their agreement on a scale of 1 to 4 (strongly disagree to strongly agree) on a variety of statements pertaining to the intervention. A response of either “agree” or “strongly agree” was defined as participant satisfaction with the intervention. In addition, a series of open‐ended questions were included to explore participants thoughts about the intervention content, their feelings about the CHW, and suggestions for improvement. The open‐ended questions were audio recorded with participant consent and translated and transcribed from Swahili to English.

### 2.9 Secondary outcomes

Secondary outcomes were measures of preliminary efficacy, including HIV care engagement, blood pressure (SBP and DBP), and hypertension knowledge.

### 2.10 Hypertension care engagement

Hypertension care engagement was measured by asking participants the following questions at their baseline and post‐intervention assessments: Since we last saw you in clinic, 1) were you seen by a doctor for your high blood pressure?, 2) did a doctor prescribe you medications for high blood pressure?, and 3) are you currently using medications for high blood pressure?. Each question was assessed separately and a positive response to any question was considered a marker of hypertension care engagement.

### 2.11 Systolic and diastolic blood pressure

Blood pressure was measured twice in the right arm with at least a 5‐minute interval between measures using an FDA‐approved automatic blood pressure monitor at the baseline assessment, all in‐person intervention sessions and at the post‐intervention assessment by either the CHW or research assistant. The two SBP and two DBP values measured at the baseline assessment and at the post‐intervention assessment were then averaged to obtain a baseline average SBP and DBP and a post‐intervention average SBP and DBP for analysis purposes.

### 2.12 Hypertension knowledge

Participants completed a survey that included questions about hypertension knowledge. The survey was orally administered by a research assistant in Swahili. Content from the survey was derived from multiple hypertension, chronic kidney disease, and cardiovascular disease surveys from SSA.^22^, ^23^, ^24^ The survey was translated from English to Swahili and then independently back‐translated to confirm fidelity to the content. The survey was administered during the baseline assessment and again at the post‐intervention assessment.

### 2.13 Data management and analysis

Data were collected on paper forms and entered into a REDCap database by a member of the research team. Data were analyzed using STATA version 16.0 (STATA Corp., College Station, TX, USA). Continuous variables were expressed using median and interquartile range (IQR). Categorical variables were expressed as frequencies.

Feasibility of the intervention was described as the proportion of eligible participants who were recruited into the study, and among those recruited, the proportion who attended all five intervention sessions and completed the post‐intervention assessment. Fidelity of the intervention was assessed by reviewing the audio‐recordings of every intervention session and examining the percentage of components from the intervention curriculum checklists that were covered by the CHW. Acceptability of the intervention was determined by examining responses to the post‐intervention patient satisfaction survey, with >80% of satisfaction used as a metric of acceptability.

Potential efficacy of the intervention was examined for all participants who completed the post‐intervention assessments. We examined whether differences existed between the baseline and post‐intervention assessments using McNemar's test for categorical variables and Wilcoxon signed rank test for continuous variables.

### 2.14 Ethics

Study procedures were approved by Duke Health Institutional Review Board (Pro00091126), Kilimanjaro Christian Medical University College Research Committee (No. 2265), and the Tanzania National Institute for Medical Research Ethics Coordinating Committee (NIMR/HQ/R.8a/Vol. IX/2779). All participants provided written informed consent prior to enrolment. Participants were reimbursed 10 000 Tanzanian shillings (approximately $4.44 in 2019 USD) at the baseline and post‐intervention assessments and 5000 Tanzanian shillings (approximately $2.22 in 2019 USD) for the other in‐person intervention assessments for cost of their time and transportation.

## 3 RESULTS

Baseline characteristics of the study sample are described in Table 2. The study enrolled 14 individuals with HIV and hypertension. The median age of participants was 54.5 (IQR 46.0 – 62.0) years and 9 (64.3%) were women. The median duration of ART use was 3.7 (IQR 3.2 – 6.8) years and median CD4 was 405 (IQR 313 – 522) cells/mm^3^. All participants had a viral load <200 copies per mL. Median BMI was 27.4 (IQR 25.1 – 28.9), and no participant was using an antihypertensive agent at the time of the baseline assessment.

**TABLE 2:** Baseline characteristics of participants enrolled in a pilot feasibility study in an HIV care and treatment center in northern Tanzania, 2019 (n = 14)

**Characteristic**	
Sex, (female), n (%)	9 (64.3%)
Age (years), median (IQR)	54.5 (46.0 – 62.0)
Marital status	
Single, n (%)	2 (14.3%)
Married, n (%)	3 (21.4%)
Separated/divorced, n (%)	5 (35.7%)
Widowed, n (%)	4 (28.6%)
Education	
None, n (%)	2 (14.3%)
Primary (grade 0 – 8), n (%)	11 (78.6%)
Secondary or higher (grade 9 or higher), n (%)	1 (7.1%)
Income per month (USD), median (IQR)	28.88 (8.88 – 66.67)
Median duration of time since HIV diagnosis, years (IQR)	4.5 (3.5 – 7.5)
Median duration of ART use, years (IQR)	3.7 (3.2 – 6.8)
CD4 current (cells/mm^3^), median (IQR)	405 (313 – 522)
Currently using antihypertensives, (yes), n (%)	0 (0.0%)
BMI, median (IQR)	27.4 (25.1 – 28.9)
Waist circumference (cm), median (IQR)	97.8 (93.5 – 100.0)

Abbreviations: IQR, interquartile range; USD, US dollars; ART, antiretroviral therapy; BMI, body mass index.

### 3.1 Feasibility, fidelity, and acceptability

**FIGURE 2:**
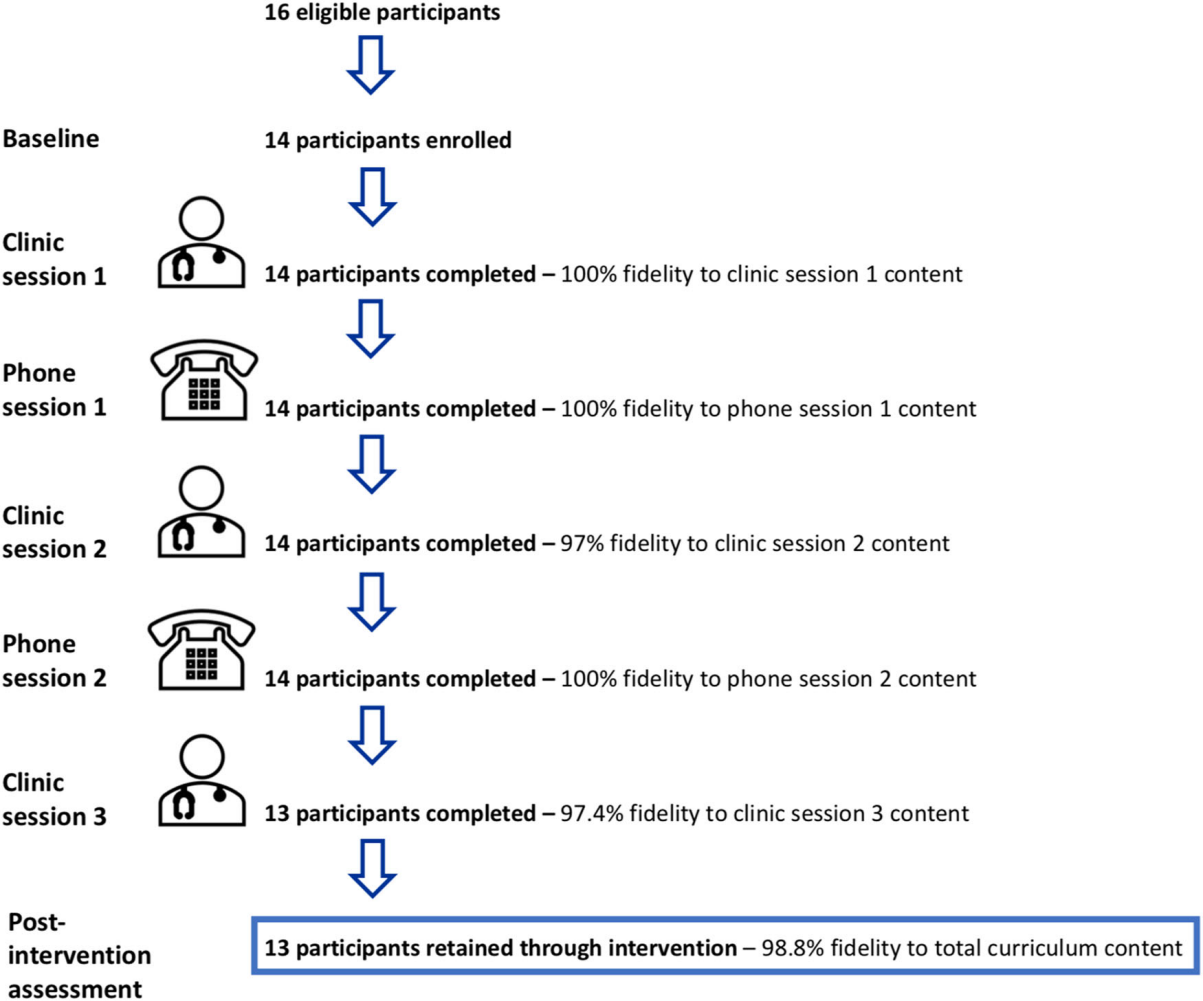
Feasibility and fidelity of community health worker‐delivered hypertension education intervention in northern Tanzania, 2019

Figure 2 describes intervention feasibility and fidelity. Based on study eligibility criteria, a total of 16 adults were eligible to participate in the study. Of these 16, one individual was not reachable by phone and another declined participation, resulting in 14 (87.5%) of all eligible individuals enrolled in the study. Among the 14 enrolled participants, 13 (92.9%) completed all five intervention sessions, including the three in‐person intervention sessions and two telephone intervention sessions. The one participant who did not complete all intervention sessions, dropped out prior to the third in‐person session, and planned to seek alternative treatment for hypertension; the participant was not interested in starting antihypertensive medications. Of the 13 participants retained through all intervention sessions, all returned for the post‐intervention assessments. Participants completed a total of 69 intervention sessions, and all intervention sessions were independently evaluated for fidelity to the intervention content. Fidelity to the intervention content was high at 98.8%.

Among participants who completed the post‐intervention assessment, feedback on the intervention was positive. All participants stated that the intervention and education materials helped them to learn about high blood pressure, and all reported that they found it helpful to speak with the CHW on the phone and to also meet with the CHW in the clinic. All participants stated that they would continue to use what they learned during the intervention to help control their blood pressure in the future. All but one (92.3%) participant reported they would recommend the intervention to a friend or family member. Regarding frequency of intervention sessions, the majority of participants reported that the number of intervention sessions was “the right amount”: 7 (53.8%) stated the number of in‐person clinic sessions was sufficient, and 10 (76.9%) stated the number of telephone sessions was sufficient. Most participants also felt that intervention length was “the right amount,” nine (69.2%) reported the length of in‐person clinic sessions was sufficient, and nine (69.2%) stated the length of telephone sessions was sufficient. Of the 13 participants, 10 (76.9%) recommended having both clinic and telephone sessions in the future, while two (15.4%) preferred to only have telephone sessions, and one (7.7%) preferred only clinic sessions.

In the open‐ended questions, participants discussed their experiences and perspectives with the intervention. All participants described appreciation of the intervention and the CHW. All participants stated that the intervention helped them to make positive changes to their lifestyle to improve their blood pressure and helped them to gain knowledge about hypertension. Frequently reported challenges about the intervention included length of the intervention sessions and financial challenges such as cost of transportation to and from the clinic, cost of antihypertensive medication, and cost of airtime when talking to the CHW on the phone if the participant initiated the call.

### 3.2 Hypertension care engagement

Engagement in hypertension care improved following the intervention. At the baseline assessment, two (15.4%) participants reported previously seeing a doctor for hypertension, compared to 11 (84.6%) participants at the post‐intervention assessment (*P* = .0027). At baseline, one (7.7%) participant reported being prescribed antihypertensive medication, compared to 10 (76.9%) participants post intervention (*P* = .0027). No participant was using antihypertensives at the start of the intervention, whereas 10 (76.9%) participants reported use of antihypertensive agents at the post‐intervention assessment (*P* = .0016).

### 3.3 Systolic and diastolic blood pressure

**FIGURE 3:**
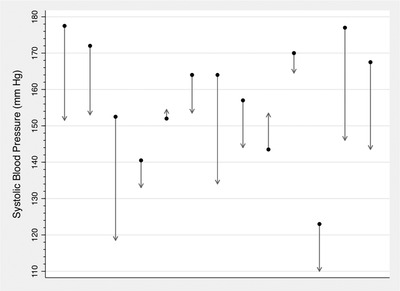
Change in systolic blood pressure from baseline to post‐intervention assessment among participants exposed to community health worker‐delivered hypertension education intervention, 2019 (n = 13)

**FIGURE 4:**
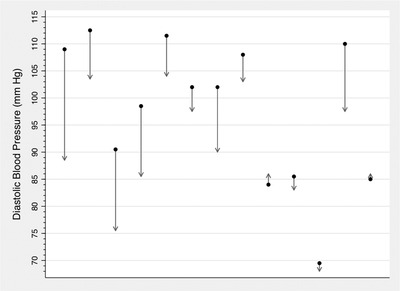
Change in diastolic blood pressure from baseline to post‐intervention assessment among participants exposed to community health worker‐delivered hypertension education intervention, 2019 (n = 13)

Pre‐intervention median SBP was 164 (IQR 152–170) mmHg, compared to post‐intervention SBP of 146 (IQR 134–154) mmHg (*P* = .0029). Pre‐intervention median DBP was 102 (IQR 86–109) mmHg, compared to post‐intervention DBP of 89 (IQR 86–98) mmHg (*P* = .0023). Changes in SBP and DBP from the time of baseline assessment to post‐intervention assessment for each participant is shown in Figures 3 and 4, respectively.

### 3.4 Changes in knowledge

There was an improvement in hypertension knowledge following the intervention. When asked “What is high blood pressure?”, no participant was able to provide a correct response at the baseline assessment. At the post‐intervention assessment, five (29.4%) participants responded correctly. At the baseline assessment, five (38.5%) participants recognized that people with hypertension did not always have symptoms, compared to eight (61.5%) participants post‐intervention (*P* = .0833). When asked, “Is having stress and worries the most common cause of high blood pressure?”, 10 (76.9%) participants responded yes at the baseline assessment, compared to six (46.2%) participants who responded yes at the post‐intervention assessment (*P* = .0588).

## 4 DISCUSSION

In this pilot feasibility study, we found that a CHW‐delivered educational intervention integrated into an HIV clinic to address hypertension was feasible and acceptable. Furthermore, our intervention resulted in significant improvements in hypertension care engagement outcomes and reductions in systolic and diastolic blood pressure. Our findings suggest that a task‐sharing intervention integrated into existing HIV care is feasible and acceptable, and shows promise in achieving engagement in hypertension care and blood pressure control.

The success of our intervention may have been reflective of an integrated model of care that leveraged existing HIV infrastructure. Our intervention was integrated into existing HIV care (i.e., all participants met with the CHW at the HIV clinic site on days they were scheduled to see their HIV provider). This was done intentionally to maximize convenience for patients and minimize additional costs related to transportation and time away from work. Furthermore, the HIV clinic often represents a safe environment where patients tend to have well‐established relationships with staff and high levels of trust in a health care system historically surrounded by mistrust and stigma.^25^ Taken together, the HIV clinic represents an opportune setting to utilize an existing platform that successfully supports retention in care to engage PLWH in the care of other chronic diseases.^26^, ^27^ Moreover, in the face of the COVID‐19 pandemic, delivery of primary care services for NCDs has been severely impacted,^28^, ^29^ and there has been a greater push towards healthcare delivery that meets the needs of PLWH. This includes providing healthcare services in a way that minimizes frequency of visits, expands access to care outside of traditional settings, and integrates care of HIV and other chronic diseases. In light of the pandemic, multiple ministries of health in SSA, with support from governmental and non‐governmental international agencies, have called for the adaptation of HIV programs to provide NCD care in order to reduce contact with health facilities and minimize risk of exposure to SARS‐CoV‐2.^30^ The findings from our study support this call to action, and provide evidence for the need to mobilize HIV clinical platforms to deliver both HIV and NCD services. Further research is necessary to test the implementation and efficacy of such models.

Our study was a pilot‐feasibility study over a 6‐week period; thus, the long‐term effectiveness of our intervention warrants further investigation. Despite this limitation, we observed encouraging results in our secondary outcomes. Notably, there was a trend towards improved hypertension knowledge among our participants. Our intervention was based on the Health Belief Model, an evidence‐based theoretical framework that emphasizes self‐efficacy and behavioral change are closely tied to increased patient awareness and knowledge of a disease.^20^, ^21^ Our previous qualitative work in northern Tanzania revealed that poor and inaccurate hypertension knowledge was the predominant barrier to hypertension engagement in PLWH.^8^ Therefore, by focusing on an intervention that targeted education we hoped to address this barrier, increase motivation and promote self‐efficacy and behavioral change, and consequently improve hypertension outcomes. Moreover, we observed substantial declines in blood pressure (approximately a 20‐point reduction in SBP and a 10‐point reduction in DBP) among our participants. Studies in similar settings have shown educational interventions improve blood pressure control,^31^, ^32^, ^33^ and we suspect that with continued delivery of our educational intervention sessions, hypertension knowledge, and blood pressure control may have increased over time in our sample.

Our results provide evidence that a task‐sharing approach utilizing a CHW may be an effective strategy in improving hypertension outcomes in PLWH. Our CHW had a non‐medical background and was trained to measure blood pressure, provide hypertension education and counseling, and refer to a prescribing provider if needed. Our CHW delivered the intervention with close to 100% fidelity, and participants in our study reported appreciation of the CHW and satisfaction with the content delivered. Prior studies in Tanzania have shown that HIV care delivery by CHWs is favorably received.^34^, ^35^ Furthermore, a few studies have also revealed that interventions offering NCD care delivered by CHWs are feasible, highly acceptable, and effective.^36^, ^37^ The utilization of CHWs to provide care may be an attractive strategy in a health care system that is overtaxed by high patient volumes and few prescribing providers.^38^, ^39^

### 4.1 Limitations

The results of our study must be interpreted in light of its limitations. First, this study was a pilot feasibility study, and thus, due to the small sample size and non‐randomized design, our study was not powered to identify statistically significant differences. In order to determine efficacy of the intervention, a fully powered randomized clinical trial is warranted in the future. Second, our study was conducted in only one HIV clinic in an urban area in northern Tanzania and so findings may not be generalizable to other settings in SSA including rural areas. Third, the post‐intervention assessment took place 2 weeks after intervention completion. In order to determine long‐term effectiveness and sustainability of the intervention, future studies should conduct the post‐intervention assessment at a later time frame.

## 5 CONCLUSIONS

Our study demonstrates that a theoretically informed, CHW‐delivered educational intervention, utilizing a task‐sharing approach and integrated into existing HIV care, is highly feasible and acceptable and has the potential to improve hypertension care engagement, increase adherence to antihypertensive medication and reduce blood pressure. Our results contribute to evidence suggesting that the existing HIV care infrastructure can be mobilized to improve care for other chronic diseases, including hypertension. Further development and testing of efficacy of our intervention is warranted across Tanzania and other similar settings.

## CONFLICTS OF INTEREST

The authors declare they have no competing interests.

## AUTHOR CONTRIBUTIONS

PM, NMT, and MHW conceived the study; PM, NMT, and MHW designed the study protocol; PM, BTM, and MHW implemented and supervised the study; PM, LW, and AM collected the study data; PM and DBM performed the data analysis; PM drafted the manuscript; PM, DBM, LW, AM, BTM, NMT, and MWH critically revised the manuscript for content. All authors read and approved the final manuscript.
